# An Efficient Method for the Synthesis of Peptoids with Mixed Lysine-type/Arginine-type Monomers and Evaluation of Their Anti-leishmanial Activity

**DOI:** 10.3791/54750

**Published:** 2016-11-02

**Authors:** Hannah L. Bolt, Paul W. Denny, Steven L. Cobb

**Affiliations:** ^1^Department of Chemistry, Durham University; ^2^School of Medicine, Pharmacy and Health, Durham University

**Keywords:** Biochemistry, Issue 117, peptoid, synthesis, arginine monomer, lysine monomer, amphipathic, parasite, Leishmaniasis

## Abstract

This protocol describes the manual solid-phase synthesis of linear peptoids that contain two differently functionalized cationic monomers. In this procedure amino functionalized 'lysine' and guanido functionalized 'arginine' peptoid monomers can be included within the same peptoid sequence. This procedure uses on-resin (*N*-(1-(4,4-dimethyl-2,6-dioxocyclohexylidene)ethyl) or Dde protection, orthogonal conditions to the Boc protection of lysine monomers. Subsequent deprotection allows an efficient on-resin guanidinylation reaction to form the arginine residues. The procedure is compatible with the commonly used submonomer method of peptoid synthesis, allowing simple peptoids to be made using common laboratory equipment and commercially available reagents. The representative synthesis, purification and characterization of two mixed peptoids is described. The evaluation of these compounds as potential anti-infectives in screening assays against *Leishmania mexicana *is also described. The protozoan parasite *L. mexicana *is a causative agent of cutaneous leishmaniasis, a neglected tropical disease that affects up to 12 million people worldwide.

**Figure:**
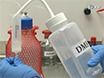


## Introduction

Peptoids (or poly-*N*-substituted glycines) are a class of peptide-mimetics that offer similar properties to peptides and as such are increasingly being investigated for medicinal and materials applications. In peptides, the side chain of each amino acid is connected to the α-carbon of the amide backbone; in peptoids the side chains are shifted onto the nitrogen atom of the backbone. Crucially, this gives peptoids greater resistance to proteolysis.

Peptoids are commonly synthesized using the submonomer method pioneered by Zuckermann *et al.,* where peptoid monomers can be built by sequential haloacetylation of an amine functionality attached to a solid support and subsequent displacement of the halogen using a primary amine.^1^ Our group has recently developed an adaptation to this submonomer method to allow lysine- and arginine-type peptoid residues to be included within the same peptoid sequence for the first time.^2^ This manual solid-phase approach to peptoid synthesis uses commercially available reagents and common laboratory equipment, making it accessible for the majority of laboratories. Peptoids have been shown to have promising activities against a wide range of Gram negative bacteria, Gram positive bacterial and fungal species that are comparable to many known antimicrobial peptides.^3-9^

In our work, peptoids have been used as novel anti-infective compounds for treatment of the neglected tropical disease leishmaniasis.^5,10^ Leishmaniasis is endemic in more than 80 countries worldwide and it is estimated that over 12 million people are infected globally.^11^ The disease is caused by protozoan parasites that are transmitted by the bite of a sandfly. *Leishmania* species can cause cutaneous leishmaniasis, a condition that leads to scarring and damage to mucous membranes, or the life-threatening visceral leishmaniasis, which causes fatal organ damage. No vaccine is currently available for this disease and existing treatments rely on a small number of drugs that have severe side effects. In addition, resistance to existing drugs is an emerging and serious problem so new treatments are desperately needed to effectively treat leishmaniasis in the future.^12-16^

In these antimicrobial applications, peptoids are often designed to be amphipathic with a mixture of cationic and hydrophobic monomers.^3,4^ This can give peptoids a degree of selectivity towards bacterial cells, reduce toxicity to mammalian cells, and to improve their activity as molecular transporters.^17-20^ The majority of the anti-infective peptoids in the literature contain cationic side chains that are exclusively comprised of either amino functionalized lysine-type monomers or arginine-type residues. Peptide-peptoid chimeras, where the cationic chains are comprised of the amino acids lysine or arginine, have also been synthesized to examine the effect of cationic groups on activity and toxicity.^21-25^

Poly-lysine peptoids can be easily synthesized using commercially available Boc-protected amines. The poly-arginine peptoids reported can be made using a method which uses pyrazole-1-carboxamidine as a guanidinylation agent.^18^ However, this can only be undertaken after the peptoid has been cleaved from the resin and Boc protection on side chains removed, so every lysine-type residue within the sequence is transformed into an arginine residue. In an effort to fine tune the chemical and biological properties of the compounds, we developed a method that allows dual cationic functionality (*e.g.*, *N*Lys and *N*Arg) to be included in any given peptoid sequence for the first time.^2^

Herein, we describe the synthesis, purification and characterization of two novel peptoids that contain both lysine- and arginine-type residues in the same sequence. The method uses orthogonal *N*-Boc and *N*-Dde protection on resin with pyrazole-1-carboxamidine as a guanidinylation reagent. The biological evaluation of these peptoids is also described in cytotoxicity assays against *Leishmania mexicana*, the causative agent of cutaneous leishmaniasis. This provides a practical method to access peptoids with dual cationic functionality and to assess their biological activity. It is expected that this method will aid the synthesis of amphipathic peptoids by the peptoid community in the future.

## Protocol

### 1. Solid-phase Synthesis of Peptoids

NOTE: Peptoids are synthesized manually using the submonomer procedure of solid-phase peptoid synthesis. This method allows high coupling efficiency and good final product yields. Synthesis on solid-phase also allows excess reactants to be removed easily at the end of each step and the method has been modified here to allow different functionalized cationic monomers (*i.e.,* arginine-type and lysine-type residues) to be included within the same sequence.^1,2^

1. **Synthesis of a linear peptoid** Caution: Carry out safety assessments before starting synthesis. Carry out all reactions in a fume hood and wear adequate personal protective equipment as appropriate (*i.e.*, disposable nitrile gloves, safety glasses and a lab coat). Take particular care when using the following reagents and solvents. Dimethylformamide (DMF) is a suspected teratogen and dichloromethane (DCM) is a carcinogen. *N,N*'-diisopropylcarbodiimide (DIC) and piperidine are hazardous to eyes, skin, via respiratory inhalation and may cause skin sensitization. Hydrazine is a suspected carcinogen, fatal if inhaled and causes severe burns to skin or eyes. Bromoacetic acid is also hazardous to skin, eyes and the respiratory tract and may cause burns upon contact. Trifluoroacetic acid (TFA) is a volatile liquid and can cause severe burns so handle with care. Heavy duty gloves are recommended.
  1. Add 0.12 g Fmoc-protected Rink Amide resin (0.1 mmol, typical loading 0.7 mmol/g) to a capped 20 ml polypropylene reaction vessel with two frits. Add 5 ml dimethylformamide (DMF) to swell the resin and leave the vessel to stand for at least 60 minutes at room temperature. Drain the DMF using a solid-phase extraction vacuum platform.
  2. To deprotect the Fmoc group on the swollen resin, add 2 ml piperidine solution (20% in DMF v/v). Place the vessel on a shaker platform at room temperature (450 rpm) and shake for 5 min. Remove the solution via vacuum station.
    1. Repeat Fmoc deprotection with 2 ml piperidine solution and shake for 15 min at room temperature. Drain the solution as before.
  3. Wash the resin by adding 2 ml DMF and mixing the resin for 30 sec. Drain the DMF and repeat three more times.
  4. For the acetylation, add 1 ml of bromoacetic acid solution (0.6 M in DMF) and 0.2 ml *N,N'*-diisopropylcarbodiimide solution (DIC, 50% in DMF v/v). Leave reaction vessel to shake for 20 min at room temperature. Drain the solution and wash the resin with 2 ml DMF three times.
  5. For the displacement, add 1 ml of amine solution (1.5 M in DMF). Shake the resin for 60 min at room temperature. Drain the solution and wash the resin with 2 ml DMF three times.
    1. To add an arginine-type monomer, follow step 1.7. Depending upon the desired peptoid sequence, different amines will be added.
  6. Repeat steps 1.1.4 and 1.1.5.
  7. To include a guanidine functionalized monomer (*i.e.*, *N*Arg), add 1 ml of unprotected diamine solution (1.5 M in DMF) to the resin and shake for 60 min at room temperature.
    1. Drain the solution and wash the resin with 2 ml DMF three times.
    2. Add 2-acetyldimedone (0.2 g, 1 mmol in 0.5 ml DMF, 10 equivalents) to add the Dde group to the free primary amine and shake for 60 min at room temperature. Drain the solution and wash the resin with 2 ml DMF three times.
  8. Continue submonomer synthesis as in 1.1.4 to 1.1.7 until the desired sequence is made. Add 2 ml DMF to wash the resin and repeat three times.
  9. To deprotect the Dde group on resin, add 4 ml 2% hydrazine solution (in DMF v/v) and shake for 3 min at room temperature. Drain the solution and repeat three times.
  10. Drain the solution and wash the resin with 2 ml DMF three times.
  11. Add pyrazole-1-carboxamidine (6 equivalents per free amine, *i.e.*, per *N*Arg monomers, in the minimum volume of DMF) and *N*,*N*-diisopropylethylamine or DIPEA (6 equivalents per free amine) and shake at room temperature for 60 min.
  12. Drain the solution and wash the resin with 2 ml dichloromethane three times. Leave the resin to dry in air for 10 min then the resin can be stored until cleavage (section 2).
  13. To pause the synthesis, wash the resin with 2 ml DMF three times. Add 2 ml DMF, stopper the synthesis vessel and leave at room temperature in a fume hood. NOTE: The synthesis may be paused after any displacement step (except the second displacement step as diketopiperazines may be formed).
2. **Side-chain deprotection and cleavage from resin**
  1. Undertake a test cleave to check the progress of synthesis (purity and mass) at any point during synthesis after the displacement steps, addition or removal of Dde-protecting group or after the final sequence has been made.
    1. Transfer approximately 10 resin beads from the reaction vessel into a new 8 ml polypropylene fritted cartridge.
    2. Add 1 ml of trifluoroacetic acid cleavage cocktail (containing 95% TFA, 2.5% H_2_O, 2.5% triisopropylsilane) and shake for 90 min at room temperature.
    3. Filter the TFA cleavage cocktail from the resin using the fritted reaction vessel into a 10 ml round bottomed flask.
    4. Evaporate the cleavage cocktail using a rotary evaporator and redissolve the resulting oil in 1 ml acetonitrile/water for submission to LC-MS or analytical HPLC.
  2. For the final cleavage: in the same fritted polypropylene reaction cartridge used for synthesis, add 4 ml of the TFA cleavage cocktail (95% TFA, 2.5% H_2_O, 2.5% triisopropylsilane) and cover the vessel. Shake for 90 min at room temperature.
  3. Filter the TFA cleavage cocktail from the resin using the fritted reaction vessel into a 50 ml round bottomed flask.
  4. Evaporate the cleavage cocktail using a rotary evaporator. After the TFA has been removed, the product should be obtained as an oil. To aid TFA removal from this crude oil, add 2 ml anhydrous diethyl ether and the peptoid should precipitate.
    1. Either remove the diethyl ether via pipette and discard or evaporate using a rotary evaporator. Repeat diethyl ether precipitation three times.
  5. Dissolve the crude peptoid in 10 ml acetonitrile/acidified water solution (50% acetonitrile, 0.1% TFA in water v/v). Transfer to a pre-weighed container, freeze at 20 °C and lyophilize to a dry powder.

### 2. Characterization and Purification

NOTE: The peptoid synthesis can be monitored and the final peptoid assessed via analytical reverse-phase HPLC using a C18 column and electrospray liquid-chromatography mass spectrometry (LC-MS). All HPLC solvents of solvents for LC-MS should be freshly prepared.

1. **Analytical HPLC**
  1. Weigh 1 mg of peptoid in a small glass vial. Add the minimum volume of acetonitrile to dissolve and dilute to 1 ml with water. Ensure that the peptoid has completely dissolved.
  2. Inject 10 µl to analytical HPLC (suggested gradient 0 - 100% solvent B over 30 min, where solvent A = 95% water, 5% acetonitrile, 0.05% TFA and solvent B = 95% acetonitrile, 5% water, 0.03% TFA), as per manufacturer's instructions.
  3. Visualize UV spectrum at 220 nm.
2. **ESI LC-MS**
  1. Make 1 mg/ml peptoid solution as in step 2.1.1.
  2. Inject 1 µl to electrospray LC-MS to determine if the molecular weight of the target peptoid is present, using manufacturer's instructions.
  3. Check the target mass of the peptoid sequence using a peptoid calculator, as per instructions on the calculator. ^26^ This web utility also allows the assignment of any deletion/addition products seen in the mass spectrum.^26^
3. **Preparative reverse phase HPLC**
  1. Dissolve crude peptoids into 2 ml acidified water/acetonitrile (95% water, 5% MeCN, 0.1% TFA) and purify by preparative RP-HPLC using manufacturer's protocol. Determine the gradient by the elution time obtained from analytical HPLC and the amount injected will depend upon the column dimensions.
  2. Visualize using a detector set at 220 nm.
  3. Collect fractions in 15 ml centrifuge tubes, freeze at 20 °C and lyophilize.
  4. Re-analyze fractions using LC-MS and analytical HPLC according to manufacturer's protocol. Recombine purified fractions.

### 3. Biological Testing against *Leishmania mexicana *Parasites

Caution: Safety assessments must be carried out before starting synthesis. *Leishmania mexicana* is classified a hazard group 2 pathogen in the U.K. and adequate control measures must be in place prior to starting testing. All work must be carried out in a class 2 microbiological safety cabinet and adequate personal protective equipment worn as appropriate (*i.e.,* nitrile gloves, safety glasses and a lab coat).

1. **Subculture of parasites**
  1. Defrost 1 ml -150 °C frozen stock *Leishmania mexicana *M379 by placing vial in a 37 °C water bath for 30 sec.
  2. Transfer the stock solution to 10 ml Schneider's Insect medium (at pH 7.0 supplemented with 15% heat-inactivated fetal bovine serum and 1% penicillin/streptomycin) in a 25 cm^3^ cell culture flask with a non-vented cap.
  3. Incubate at 26 °C for 72 hr.
  4. Examine parasites under microscope (400X magnification) to check condition. They should be insect stage promastigotes, procyclic forms in log phase with many dividing cells.
  5. Maintain procyclic promastigotes by sub-culturing parasites to a concentration of 5 x 10^5^ parasites/ml every three days. Count cells using a Neubauer improved hemocytometer.
2. **Transformation of* L. mexicana* insect stage into mammalian stage axenic amastigote form ^27^**
  1. Day 0: Prepare 10 ml culture of log stage parasites at 5 x 10^5^ parasites/ml in Schneider's Insect medium (at pH 7.0 supplemented with 15% heat-inactivated fetal bovine serum and 1% penicillin/streptomycin) in a 25 cm^3^ cell culture flask with a non-vented cap.
  2. Incubate at 26 °C for 48 hr.
  3. Day 3: Transfer 10 ml culture to a 50 ml centrifuge tube and centrifuge at 447 x g for 5 min.
  4. Pour off old medium and add 10 ml Schneider's Insect medium (at pH 5.5 supplemented with 20% heat-inactivated fetal bovine serum and 1% penicillin/streptomycin). Gently resuspend the pellet of parasites in the new medium using a pipette.
  5. Count the number of parasites using a Neubauer improved hemocytometer. Dilute to a concentration of 5 x 10^5^ parasites/ml with pH 5.5 medium and transfer to a 25 cm^3^ cell culture flask.
  6. Incubate at 26 °C for approximately 6 days.
  7. Day 9: Examine parasites under a microscope (400X). They should be in the non-replicating, infectious metacyclic promastigote stage.
  8. Count the number of parasites using a Neubauer improved hemocytometer. Dilute to a concentration of 5 x 10^5^ parasites/ml with pH 5.5 medium.
  9. Remove 10 ml of cell culture and transfer to cell culture flask. Incubate at 32 °C for 5 days.
  10. Day 14: Check appearance of parasites under the microscope (400X). They should be in the pathogenic amastigote stage, lacking the flagellum characteristic of promastigotes, and ready for assay.
3. **Cytotoxicity assay on *L. mexicana* axenic amastigotes.** NOTE: The biological testing of peptoids uses high-throughput assays carried out in 96 well plates. This protocol describes the testing of *L. mexicana *axenic amastigotes, but identical assays can also be carried out on the promastigote stage parasites in appropriate media. Compounds are incubated with parasites at concentrations from 3 - 100 µM for 60 min and then incubated for 24 hr following a 10-fold dilution. Results are gained by measuring the fluorescence of wells after incubation with Resazurin-based cell viability solution (*e.g.*, alamarBlue).
  1. Prepare compound stock solutions. Weigh out 1 mg of the final purified peptoid product using an analytical balance. Add the appropriate volume of molecular biology grade dimethylsulphoxide (DMSO) to a concentration of 5 mM. Make 6 µl aliquots and freeze at -20 °C.
  2. Prepare compound solutions on 96 well plates (a recommended plate layout can be seen in the results section, **Figure 6**) in triplicate from 100 to 3 µM. Add 2 µl of 5 mM stock solution of each compound in the top row (*i.e.,* A). Add 48 µl fresh Schneider's Insect medium (at pH 5.5 and 20% fetal bovine serum (FBS), 1% penicillin/streptomycin(P/S)) to top row using a multi-channel pipette. Add 25 µl Schneider's Insect medium to all other rows (B - F).
    1. Carry out a serial dilution by pipetting 25 µl solution from the top row. Add to the row below and mix. Carry out dilutions until the last row, where the last 25 µl solution should be discarded.
    2. Use amphotericin B (5 mM stocks) as a positive control and DMSO (2% solution) as a negative control in triplicate.
  3. Prepare parasite solution: transfer culture to a 50 ml centrifuge tube and centrifuge at 447 x g for 5 min. Pour off old medium and add 10 ml Schneider's Insect medium (at pH 5.5 and 20% FBS, 1% P/S).
  4. Gently dissolve the pellet of parasites in the new medium using a pipette and count using a Neubauer improved hemocytometer. Dilute culture to 8 x 10^6^ parasites/ml.
  5. Add 25 µl *L. mexicana *culture to each well. Incubate plates for 60 min at 32 °C.
  6. Remove plate from the incubator and remove 40 µl solution from each well.
  7. Add 90 µl fresh medium and incubate for 24 hr at 32 °C.
  8. Add 10 µl Resazurin-based cell viability solution to each well. Incubate for 4 hr at 32 °C.
  9. Measure the fluorescence using a plate reader (λ_ex_ = 540 nm, λ_em_ = 600 nm), as per manufacturer's instructions. Analyze data by removing average background (from medium only wells) and comparing the fluorescence of wells after normalization with respect to the DMSO controls.

## Representative Results

As a representative result, the synthesis and characterization of two 12 residue peptoids containing two lysine-type monomers and two-arginine type monomers each will be described. The subsequent results from cytotoxicity assay are also shown.

Two peptoids [(*N*nArg*N*spe*N*spe)(*N*ae*N*spe*N*spe)]_2 _(a) and [(*N*hArg*N*spe*N*spe)(*N*Lys*N*spe*N*spe)]_2 _(b) were synthesized using 120 mg Rink Amide resin each (loading = 0.79 mmol/g). All acetylation and displacement steps were carried out as described above, with all reagents purchased commercially. For these residues, the following amines were used in the displacement step: ***N*****spe** (S)-(-)-α-methylbenzylamine, ***N*****ae ***N*-(*tert*-butoxycarbonyl)-1,2-diaminoethane, ***N*****Lys*** N*-(*tert*-butoxycarbonyl)-1,4-diaminobutane. For the arginine derivative residues, the following unprotected diamines were coupled: ***N*****hArg** 1,4-diaminobutane or ***N*****nArg** 1,2-diaminoethane, followed by on-resin protection with 2-acetyldimedone (Dde-OH). After the entire sequence had been synthesized, hydrazine deprotection of the Dde yields free amines to guanidinylate.


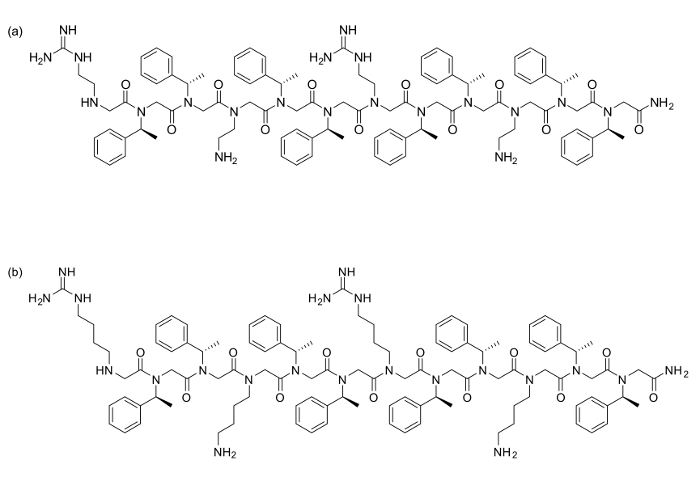
 **Figure 1. Peptoid structures (a) [(*N*nArg*N*spe*N*spe)(*N*ae*N*spe*N*spe)]_2 _and (b) [(** ***N*** **hArg** ***N*** **spe** ***N*** **spe)(** ***N*** **Lys** ***N*** **spe** ***N*** **spe)]** **_2_.** Please click here to view a larger version of this figure.


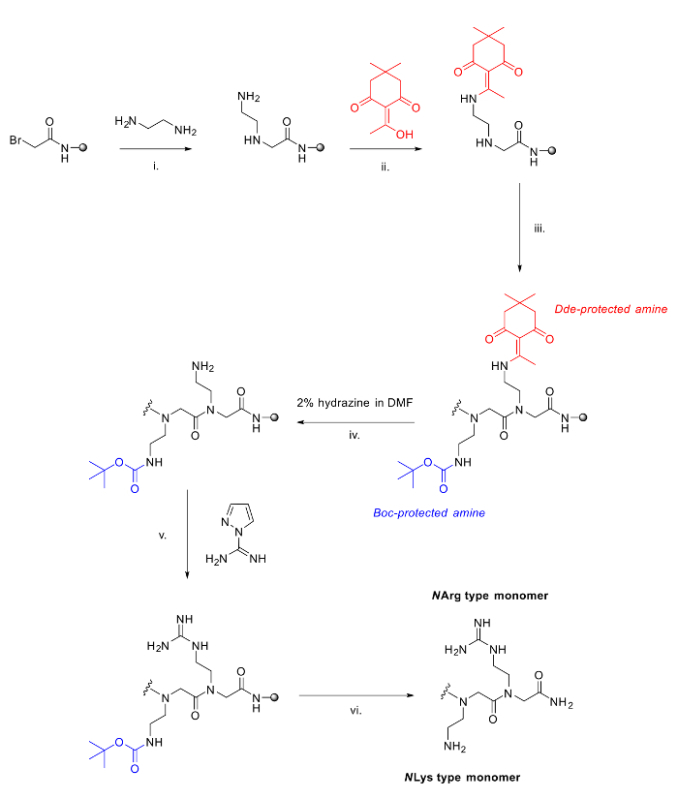
**Figure 2. The method used to synthesize mixed arginine/lysine peptoids.** i. Standard displacement step in the submonomer method with diamine; ii. Addition of Dde-OH, 90 minutes to protect free amine; iii. Further additions to extend the peptoid chain using the submonomer method; iv. Deprotection of Dde using 2% hydrazine in DMF; v. Guanidinylation of free amine on resin with pyrazole-1-carboxamidine and DIPEA in DMF; vi. Acidic cleavage from the resin and deprotection of Boc groups. Please click here to view a larger version of this figure.

Following cleavage from the resin and lyophilization, the crude products were obtained as white powders: (a) 154 mg, (b) 163 mg. Products were purified via RP-HPLC as described with maximum 50 mg injections using a LC pump with a UV-vis detector (λ = 250 nm) on an analytical column, 250 mm x 10 mm, 5 µm; flow rate = 2 ml/min. Fractions corresponding to the target mass were combined and obtained as white powders: (a) 54 mg (b) 65 mg, final yields of approximately 30% for fractions >90% pure.

The final compound identities after purification were confirmed by LC-MS (see **Figure 3**) using a triple quadrupole mass spectrometer equipped with an UPLC and a photodiode array detector. Accurate mass spectrometry was undertaken using the same spectrometer on the [M+2H]^2+^ ions. The following calculated and observed masses were found in close agreement (**Figure 4**): (a) calculated = 896.0026 amu, observed = 896.0038 amu; (b) calculated = 952.0691 amu, observed = 952.0730 amu.


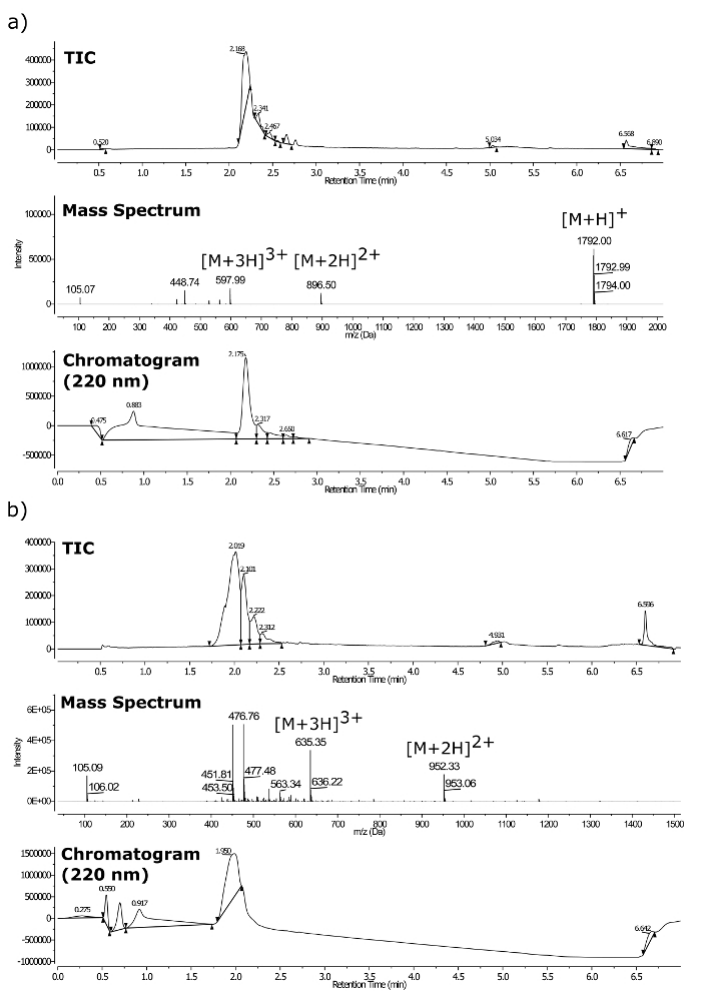
**Figure 3. LC-MS for purified peptoids.** (**a**) *m*/*z* = 1,792 and (**b**) *m*/*z* = 1,903. Where the top is TIC, middle LC-MS spectrum, bottom UV chromatogram at 220 nm. Please click here to view a larger version of this figure.


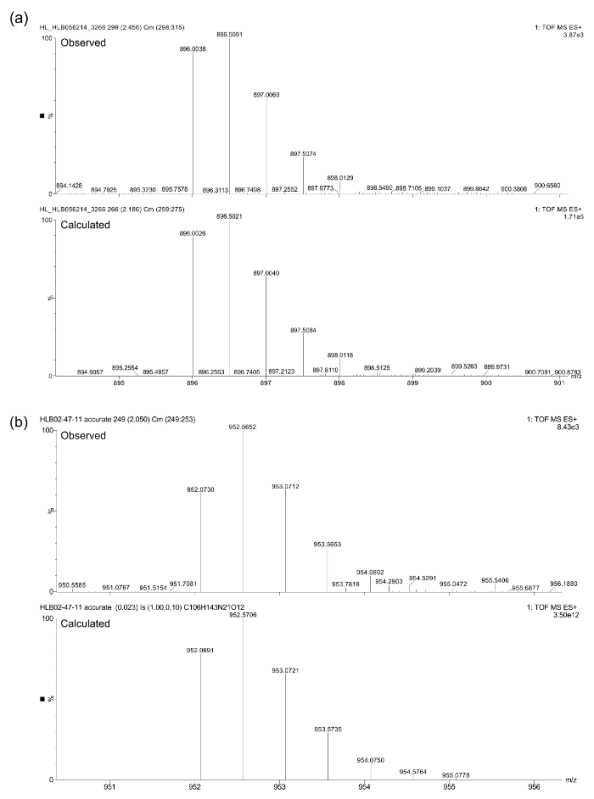
 **Figure 4. Accurate mass spectrometry data for peptoids (a) and (b).** Please click here to view a larger version of this figure.

Product purity was assessed using an analytical RP-HPLC (LC pump with a UV-vis detector on an analytical column, 4.6 mm x 100 mm, 3.5 µm; flow rate = 1 ml/min), and visualized at 220 nm, the absorbance of amide backbone. **Figure 5a **and** 5b** shows that the compounds are homogeneous.


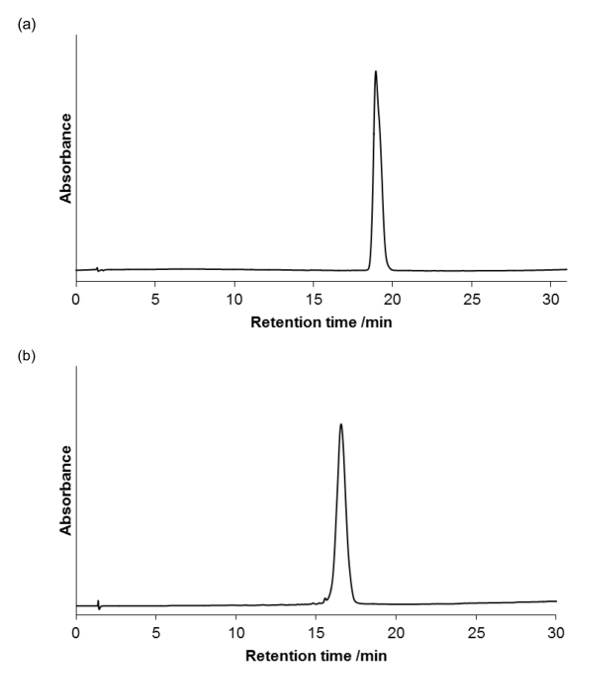
**Figure 5. Analytical HPLC for the purified peptoids (a) and (b).** There is a gradient 0-100% B over 30 minutes, column oven at 40 °C (A = 95% H_2_O, 5% MeCN, 0.05% TFA; B = 95% MeCN, 5% H_2_O, 0.03% TFA). Please click here to view a larger version of this figure.

The purified peptoids (a) and (b) were tested in cytotoxicity assays against* L. mexicana* axenic amastigotes. Frozen stocks of *L. mexicana *were defrosted and transformed to the amastigote stage ready for the assay. 72 hours after defrosting, the parasites should be insect stage promastigotes in their procyclic form, in log phase with many dividing cells. At this stage the parasites can be transformed into the amastigote stage using the pH and temperature shift described.^27^ At Day 9 of the transformation, the parasites will be in the non-replicating infectious metacyclic promastigote stage. Finally at Day 14, the parasites should be in the pathogenic amastigote stage where parasites lack the characteristic flagella of the promastigotes.^28^

5 mM stock solutions of the compounds were made in cell culture grade DMSO and tested in triplicates on a minimum of two occasions to ensure a robust data set was collected. A representative 96-well plate plan is shown in **Figure 6**. At the end of the assay, the cell viability reagent was added to each well and the fluorescence was measured as described to calculate the viability of parasites at each concentration tested (see **Figure 7**). ED_50_ values were calculated as peptoid (a) >100 µM and peptoid (b) 37 µM respectively. The error bars plotted, show the variation between wells as a standard deviation and it can be seen that these are reasonable for most bars.


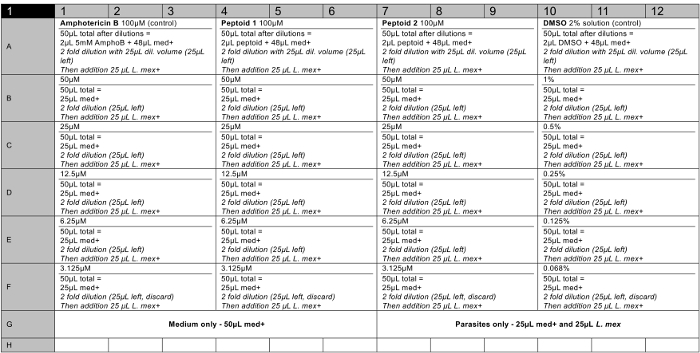
**Figure 6. Representative 96 well plate plan for a cytotoxicity assay on 2 peptoid solutions (including positive and negative controls).** med+ = medium (Schneider's Insect Medium, pH 5.5, 20% FBS, 1% P/S). *L. mex*. = 8 x 10^6^/ml parasite culture in med+. AmphoB = 5 mM in DMSO. Peptoid = 5 mM stock solution in DMSO. Empty wells should contain sterile water. Please click here to view a larger version of this figure.


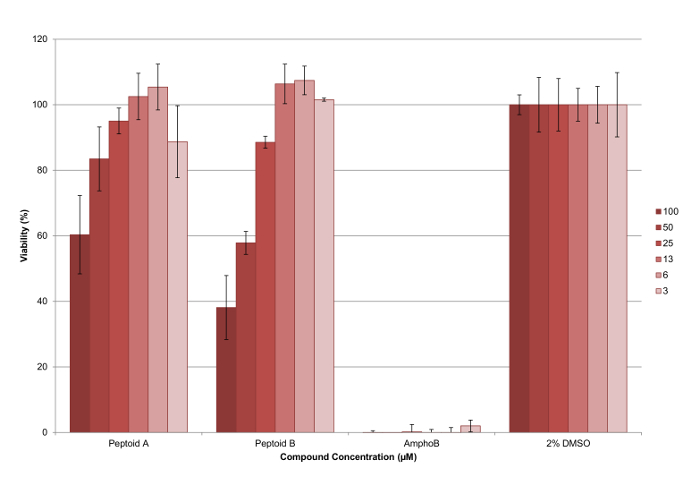
**Figure 7. Results from cytotoxicity assay against *L. mexicana *axenic amastigote parasites using peptoid (a) and (b).** It can be seen that the peptoid (b) is more effective than peptoid (a) at reducing the percentage of viable parasites, with both compounds having a dose dependent effect. The error bars plotted show the variation between wells as a standard deviation. Please click here to view a larger version of this figure.

## Discussion

Peptoids are increasingly being studied within the chemical biology and medicinal chemistry fields in applications such as novel therapeutics^3-5^, cell delivery agents^18,20^ and diagnostic tools.^29^ Typically, these sequences are cationic to provide a degree of selectivity for the pathogen over mammalian cells, the ability to penetrate through cell membranes and also aid solubility in aqueous systems. There are numerous examples of cationic peptoids that contain solely lysine- or arginine-mimetic residues in the literature. However, to date, the synthesis of peptoids that contain both of these cationic residues in the same sequence has been hindered by lack of a suitable synthetic procedure. The protocol described here allows mixed cationic peptoids to be synthesized in an efficient manner and is highly desirable as it offers a route to modulate the biological and chemical properties of amphipathic peptoids.

Our method uses an adaptation to the commonly used submonomer peptoid synthesis and permits the addition of both lysine- and arginine-type monomers within the same sequence. It uses room temperature couplings and established protecting group chemistry so it is anticipated that this method will be useful for the majority of research groups. To add orthogonal protection for the arginine-type residue, an unprotected diamine is added under standard displacement conditions and then protected in a 60-minute coupling with Dde-OH. A variety of diamines can be used which allows side chains from 2 carbons to 6 carbons long to be installed, *i.e.,* 1,2-diaminoethane to 1,6-diaminohexane respectively. The protecting Dde-OH dissolves well in DMF and is a very efficient and selective protecting group for primary amines. The Dde-protection group leaves secondary amines unaffected, *e.g.*, the unprotected N terminus of peptoid chain.^30^ One limitation is that all of the synthesis was undertaken manually; however, it is anticipated that the coupling conditions developed make the method amenable for use with automated peptide/peptoid synthesizers.

The on resin deprotection of the Dde-groups is undertaken using a 2% hydrazine solution in DMF to leave free amines that can be guanidinylated on resin using pyrazole-1-carboxamidine. Six equivalents of the pyrazole-1-carboxamidine and six equivalents of DIPEA are used per free amine on the resin (*i.e.*, six equivalents for each *N*Arg type monomer to be installed). Again, this reaction is also efficient and the pyrazole-1-carboxamidine reagent has good solubility in DMF. Completion of the reaction is typically seen via LC-MS after 60 minutes at room temperature.

Due to the versatility of the submonomer method, a wide variety of primary amines can be used in the displacement step so conditions may need to be optimized to increase coupling efficiency and overall product yields or purity.^31^ For the sequences discussed above, no special conditions were necessary for successful couplings. However, longer displacement times or higher amine concentrations could be used for problematic displacements (*i.e.*, for poorly nucleophilic or sterically bulky amines). Some amines may not be completely soluble in DMF, in which case it is recommended to dissolve these in *N*-methyl-2-pyrrolidone (NMP), or other appropriate solvents for solid-phase reactions instead as in a previous comprehensive method for submonomer synthesis of peptoids.^32^ To incorporate monomers that contain unprotected heteroatoms in the side chains, acetylation using chloroacetic acid has been shown to be effective by other groups.^33^ Additionally, other resins may be used with this method to yield peptoids with different C terminal functionalization. Wang resins and 2-chlorotrityl chloride resins are routinely used in the submonomer synthesis of peptoids. For example, this method has been successfully used with 2-chlorotrityl chloride resin in our group.^2^ Different solid supports will require a different loading procedure to Rink Amide discussed here (dependent on the specific resin used) so this should be checked with the literature prior to synthesis.

Similar to peptide synthesis, the conditions for final cleavage of the peptoid off resin can also be optimized for the specific sequence. In this protocol, a TFA cleavage cocktail was used (with triisopropylsilane and water as scavengers). The peptoids presented here contained only Boc protection which is a reasonably acid labile group. To ensure complete deprotection of sequences with a greater proportion of protected residues or less acid labile protecting groups, longer cleavage times may be necessary (*i.e.*, cleavage times in excess of 2 hours are recommended for sequences that contain Pbf or tertiary-butyl ester protected groups). Alternative scavengers can also be used for specialized side chains (for example, ethanedithiol or 2-mercaptoethanol are often used in peptides that have sulphur containing side chains like cysteine or methionine).

The biological assay presented is a standard cytotoxicity test which can be altered to suit different cell lines. It is important to note that each 96-well plate should contain sufficient controls to allow confidence in the results obtained. In this case, amphotericin B is used as a positive control as it is a known drug used to treat the disease and DMSO is used as a negative control as this is the solvent used to make compound stocks for the assay. If other cell lines are being used, alternative, suitable controls should be obtained and validated before use. *L. mexicana *is incubated with the peptoid at concentrations between 100 and 2 µM for one hour, and then the parasite/peptoid solution is diluted by a factor of ten for overnight incubation (*i.e.,* wells initially with 100 µM peptoid stock are diluted to 10 µM).

The cell viability reagent is added to each well (10% of total well volume) at the end of the assay. A visible color change is seen between wells with viable parasites (pink), control wells with no viable parasites (blue) and a spectrum between with intermediate numbers of viable parasites. The fluorescence is proportional to the number of living cells and corresponds to the metabolic activity of the cells; resazurin dye (non-fluorescent) is converted to the fluorescent resorufin by reduction reactions in metabolically active cells.^34^ In this assay, the incubation time with the cell viability reagent has been optimized for *L. mexicana*. Incubation times with the viability reagent will vary for plates seeded at different cell concentrations or with different cell lines (for example this method can also be used with mammalian cells). Dependent on the exact plate reader used, considerations need to be made before fluorescence measurements are taken. Any air bubbles in wells should be removed to ensure accurate readings can be taken. Some plate readers read from the bottom of plates, in which case flat bottomed 96 well plates should be used. Other machines may read from the top of the plate, so the lid of the plate should be removed before measurement.

Finally, in the future, this protocol may be compatible with automated synthesizers that are capable of making many sequences in parallel. Additionally, the synthesis of cyclic peptoids is also possible using this method. This protocol should provide researchers with a practical synthetic procedure that can be used to access novel peptoid scaffolds with both lysine- and arginine-type monomers, which may be of use in many applications, including materials or medicinal fields.

## Disclosures

The authors have nothing to disclose.
